# Biomechanical evaluation of a novel correction technique for adolescent idiopathic scoliosis: finite element analysis

**DOI:** 10.3389/fbioe.2025.1692204

**Published:** 2025-11-24

**Authors:** Anli Shi, Rong Ma, Zhiqiang Wang, Xiaoyin Liu, Jianqun Zhang, Zhaohui Ge

**Affiliations:** 1 Department of Orthopaedics, The First Clinical Medical College of Ningxia Medical University, Yinchuan, China; 2 Department of Orthopaedics, Gansu Provincial Hospital, Lanzhou, China; 3 Department of Orthopaedics, Henan Provincial Hospital, Zhengzhou, China

**Keywords:** novel surgery, derotation, biomechanics, complication, degeneration

## Abstract

**Purpose:**

To introduce a novel surgical technique, the Simultaneous Double-Rod Reverse Derotation (SDRRD), and to compare its corrective outcomes and biomechanical performance with traditional surgery using finite element analysis.

**Materials and methods:**

Three finite element models, including the single-rod fixed pedicle screw model (M1), the double-rod polyaxial pedicle screw model (M2), and the double-rod monoplane pedicle screw model (M3) were constructed using CT data from a Lenke 1 C N adolescent idiopathic scoliosis patient. The rod rotation technique was applied in M1 and M2, while the SDRRD technique was implemented in M3. The surgical outcomes in three dimensions and the biomechanical outcomes in the spine and implants were investigated following the simulation of the corrective process.

**Result:**

Regarding correction efficacy, M2 and M3 overmatched M1 in the coronal and sagittal planes, while M3 achieved superior axial rotation correction compared to M1 and M2. The stress on the apical vertebra is most considerable, while that on the lower instrumented vertebra is lowest in the vertebrae and intervertebral discs across M1 to M3. A considerable stress reduction is observed from M1 to M3 in most regions, with the highest stress in M1 and the lowest in M3. There is no considerable difference in the maximum stress of the implants among the three groups.

**Conclusion:**

The SDRRD technique yields favourable outcomes in coronal and sagittal plane correction, particularly in reducing axial rotation. Moreover, SDRRD effectively minimizes the stress experienced by the vertebrae and intervertebral discs.

## Introduction

Adolescent idiopathic scoliosis (AIS) is defined as a three-dimensional deformity including the structural curve of the spine in coronal and sagittal planes as well as axial rotation (Cheng et al., 2015). While the exact pathogenesis is not clear, it can generate various appearances comprising cosmetic abnormalities and physical disorders (Peng et al., 2020). Corrective surgery which should achieve the prevention of scoliosis progression, restoration of spinal alignment and balance of adverse outcomes is recommended for patients with severe curves (Coe et al., 2006; Weinstein et al., 2008). However, with the enormous evolution of implants and reduction techniques during the past, there is no single approach that can thoroughly reduce spinal deformity in three dimensions (Miller et al., 2020).

The Rod Derotation (RD) technique, which entails rotating the pre-bent connecting rod on the concave side by 90°, is designed to transform the coronal plane deformity into a sagittal plane curvature. Unfortunately, it may inadvertently extend and magnify the coronal plane deformity into the sagittal plane, thereby worsening the rib hump (Dubousset and Cotrel, 1991). The Simultaneous Double-Rod Rotation correction technique, involving the placement of pre-bent rods on both the concave and convex sides and the rotation of the concave-side rod to facilitate the spontaneous rotation of the convex-side rod, has been shown to enhance sagittal plane correction (Ito et al., 2010; Sudo et al., 2014). With the maturation of correction techniques in the coronal and sagittal planes, the development of methods for correcting axial plane rotational deformities has also continued to progress. While the Direct Vertebral Rotation (DVR) technique effectively corrects axial deformities by rotating vertebrae in the opposite direction using one or more pairs of screws, it also poses risks including pedicle screw loosening and potential nerve root damage (Lee et al., 2004; Potaczek et al., 2009). With the advancement of internal fixation devices, the application of new types of pedicle screws can also produce different correction effects. Research indicates that monoaxial pedicle screws are more effective in correcting derotational deformities and thoracic asymmetry in AIS patients with primary thoracic curves when the coronal correction outcomes are comparable (Kuklo et al., 2005).

**FIGURE 1:**
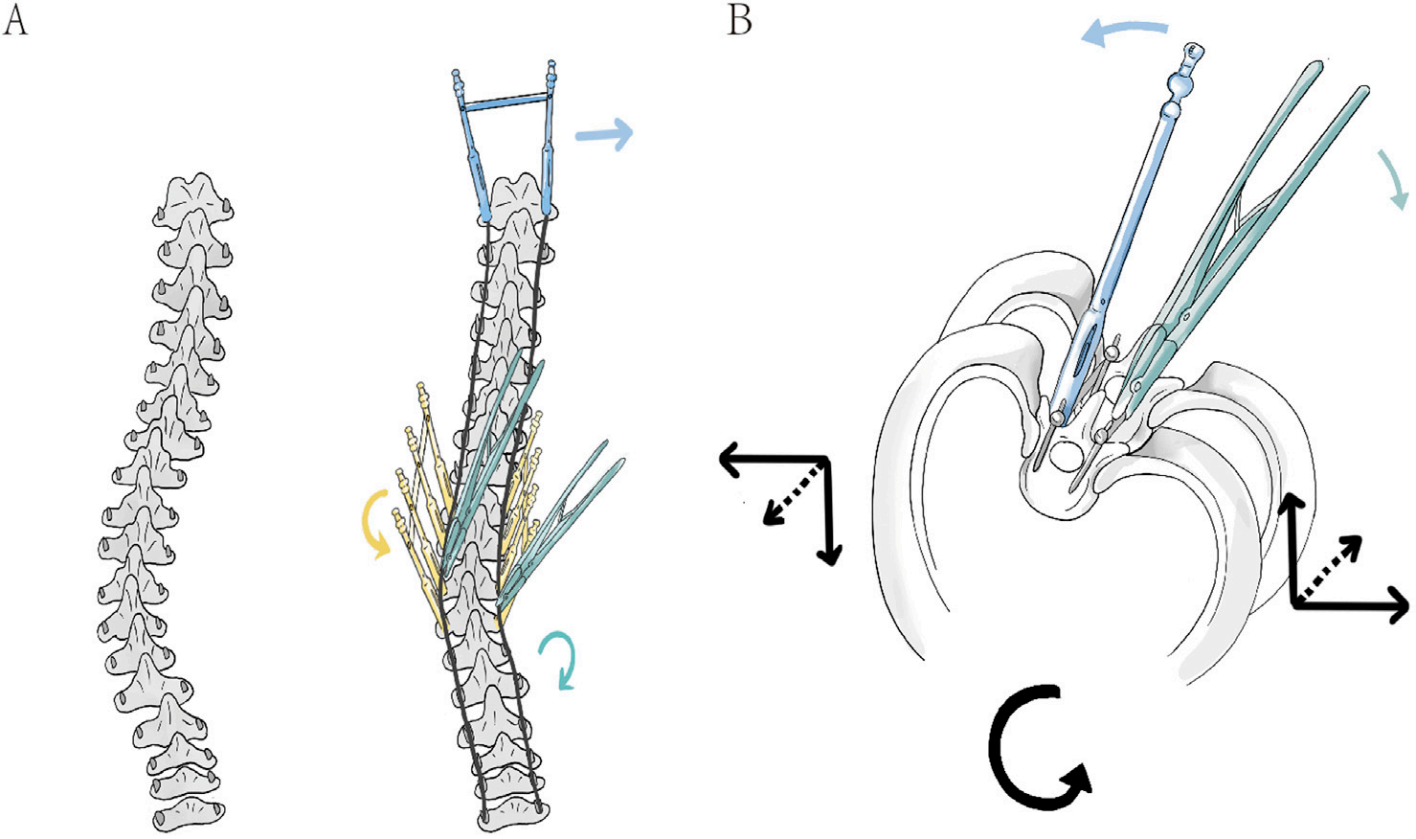
**(A)** In the coronal plane, the connecting rod is pre-bent to the anticipated correction curvature and inserted into the screws to compress the screw-vertebra complex, thereby achieving an initial reduction of the Cobb angle (right). And then, the application of three types of corrective forces is shown: the green rod rotation clamp represents simultaneous double-rod rotation correction (green arrow), the yellow fixed sleeve represents counter-rotation correction of vertebral rotation in the apical region (yellow arrow), and the blue fixed sleeve represents LIV fixation to counteract rotation (blue arrow). **(B)** In the axial view, the correction in the apical region is demonstrated: the green double rods apply a clockwise rotational corrective force upward and to the right (green arrow), while the blue sleeve applies a anticlockwise counter-rotational force downward and to the left (blue arrow). The combined force in the axial plane corrects the vertebrae in the direction opposite to the initial deformity rotation (black arrow).

Therefore, to harness the strengths of the previously discussed traditional correction techniques, a novel surgical correction method has been proposed: the Simultaneous Double-Rod Reverse Derotation (SDRRD) technique (Figure 1). The SDRRD technique is designed to correct coronal and sagittal deformities while simultaneously addressing apical vertebral rotation. It aims to mitigate the risk of increased sagittal malalignment associated with pure RD and reduce potential complications linked with DVR. Based on our team’s experience, this technique can achieve satisfactory correction outcomes in patients with AIS. To validate the biomechanical advantages of the new technique, a finite element analysis (FEA) similar to that employed in previous studies (Wang et al., 2019; Dumas et al., 2005) was conducted to compare the SDRRD technique with conventional methods in terms of three-dimensional correction outcomes, as well as the biomechanical behavior of both the scoliotic spine and the implants.

## Materials and methods

### Participant acquisition

A 13-year-old female participant (Height: 145.42 cm, Weight: 42.26 kg) with Lenke 1 C N (Lumbar Spine Modifier C is used when the center sacral vertical line falls completely medial to the entire concave lateral aspect of the thoracolumbar or lumbar apical vertebral body or bodies; Sagittal Thoracic Modifier N refers to a thoracic kyphosis (T5–T12) measuring between +10° and +40°) classification of AIS was enrolled in this study. The primary radiographic parameters included a Cobb angle of 43°, Apical Vertebral Translation (AVT) of 36.68 mm, Thoracic Kyphosis (TK) of 30.02°, Lumbar Lordosis (LL) of −52.35°, and Apical Vertebral Rotation (AVR) of 26.8° according to RAsag (the angle of rotation of a vertebra about the longitudinal axis relative to the sagittal plane) (Aaro and Dahlborn, 1981). The entire curve spanned from T4 to L3, with the major curve oriented to the right. The participant underwent a preoperative full-spine CT (GE, US) scan as well as supine preoperative supine anteroposterior and lateral full-length spinal radiographs. The participant had no history of prior surgery, nor did she have congenital scoliosis or neurogenic scoliosis. This study was approved by the Institutional Ethics Committee of Ningxia Medical University (Approval No. KYLL-2022-1137), and written informed consent was obtained from her guardian.

### Establishment of a three-dimensional model of the scoliotic spine

All CT images were saved in Digital Imaging and Communications in Medicine format by scanning in 0.625 mm slices and then imported to Mimics Research 20.0 (Materialise, Belgium) to extract segmented vertebrae by Region Growing and Edit Mask. Only T1 to sacrum were extracted to simplify the model and reduce the calculated errors. For vertebrae with rough surfaces, the surface was further smoothed, and the entire vertebra model was negatively offset by 1 mm inward using Geomagic Wrap 2017 (Raindrop, US), with the pedicle removed to simulate cancellous bone while retaining the original surface to simulate cortical bone (Chen et al., 2021). Subsequently, every smooth cortical and cancellous surface was individually formed solid substance while the annulus fibrosis, nucleus pulposus, endplate and articular cartilage were also established in Solidworks 2018 (Dassault Systemes, US) and eventually assembled into a complete scoliotic spine model.

### Construction of surgery model

**FIGURE 2:**
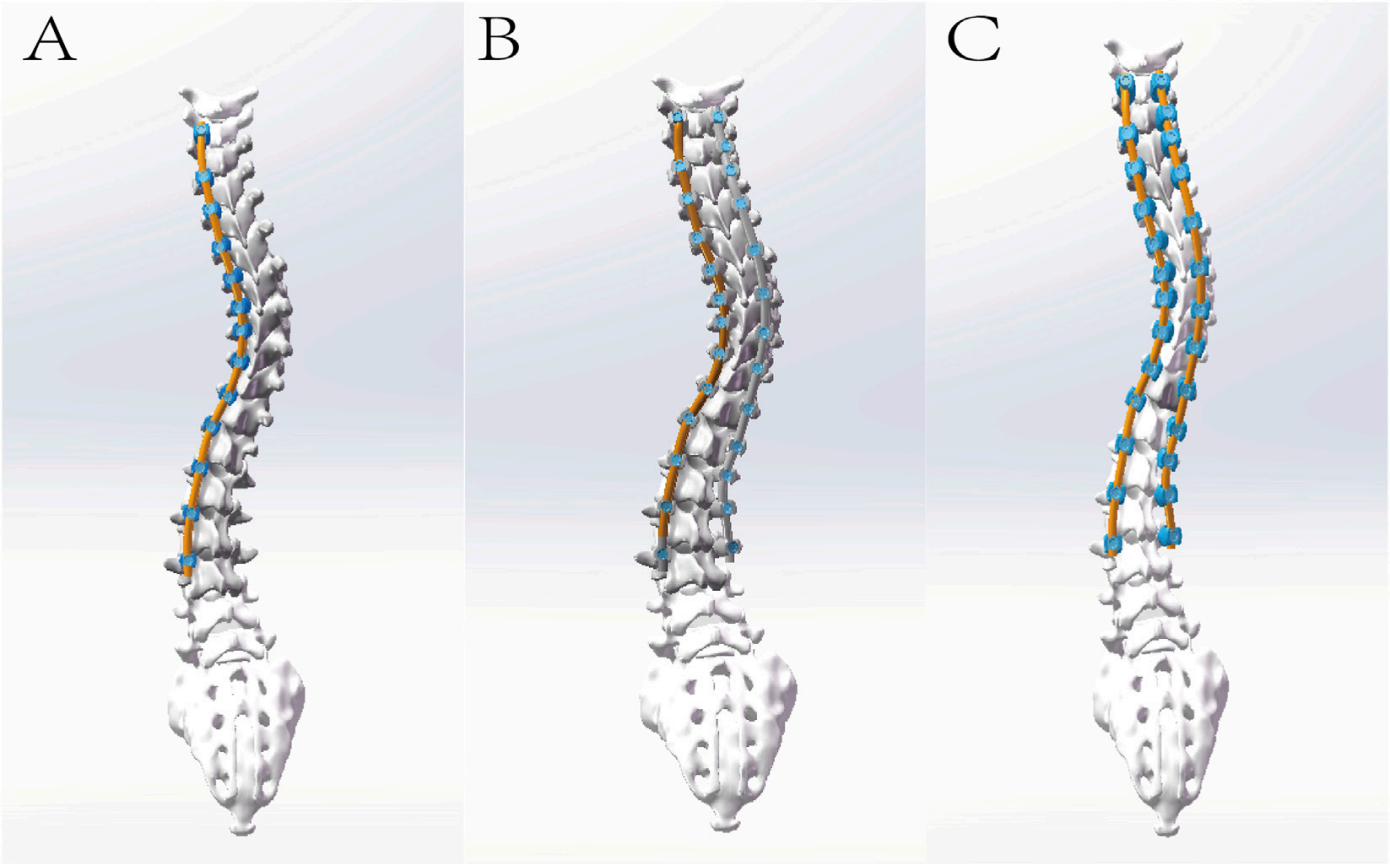
The illustration depicts the following models, each incorporating intervertebral discs and facet joint cartilage: **(A)** Single-rod fixed pedicle screw model (M1); **(B)** Double-rod polyaxial pedicle screw model (M2); and **(C)** Double-rod monoplane pedicle screw model (M3). Meanwhile, the cobb angle of M3 has been reduced for simulation of initial reduction of the Cobb angle by pre-bent rod.

Fixed pedicle screw models, monoplane pedicle screw models, and polyaxial pedicle screw models were initially created. To avoid excessive computational demands, the screws were modelled without threads and individually selected based on the pedicle diameter and vertebral length to ensure they did not penetrate the pedicle wall. A 5.5 mm connecting rod was designed by connecting the centers of the screw heads. Subsequently, the screws were assembled to form three distinct models: a concave single-rod fixed pedicle screw model (M1), a double-rod polyaxial pedicle screw model (M2), and a double-rod monoplane pedicle screw model (M3). The screws were placed in a full-segmental fashion on both the concave and convex sides, with the fixation spanning from T3 to L3. For M3, the screws were installed to allow movement in the sagittal plane. The primary objective of the finite element analysis in this study was to compare the forces experienced by the vertebrae and intervertebral discs during different rod rotation processes. However, it was not feasible to replicate the original translational process in the design—where the pre-bent rod, with curvature less than the original Cobb angle, compresses the screw-vertebra complex towards the rod during the rod compression process. Therefore, when setting up the connecting rod for M3, the original Cobb angle of the vertebrae was already reduced by translation (Figure 2).

### Conversion of the finite element model

Finite element analysis of three surgery models was performed by Ansys Workbench 17.0 (ANSYS, US). The surgical models were meshed in the form of tetrahedral grids using Mechanical. The anterior longitudinal ligament, posterior longitudinal ligament, ligamentum flavum, interspinous ligament, supraspinous ligament, and transverse process ligament were modelled using springs to simulate their mechanical properties. The contact surfaces between the annulus fibrosus and nucleus pulposus of the intervertebral disc, the endplate and the vertebra, and the endplate with the annulus fibrosus and nucleus pulposus were defined as bonded constraints. For the articular cartilage and facet joint connections, one side was set as non-separable, while the other side was defined as a bonded constraint. The contact surfaces between the screws and the vertebrae were also set as bonded constraints. The interface between the screws and the rod was defined as frictional, with a friction coefficient of 0.2. The material properties in this model are shown in Table 1 (Zhang et al., 2021).

**TABLE 1:** Material parameters of spinal model.

Tissues	Young’s modulus (Mpa)	Poisson ratio
Cortical bone	12000	0.3
Cancellous bone	100	0.3
Endplate	25	0.25
Annulus fibrosis	4.2	0.453
Nucleus pulposus	1	0.499
Articular cartilage	50	0.3
Titanium alloy	110000	0.25
Anterior longitudinal ligament	20	0.3
Posterior longitudinal ligament	20	0.3
Ligamentum flavum	19.5	0.3
Interspinous ligament	58.7	0.3
Supraspinous ligament	15	0.3
Intertransverse ligament	11.6	0.3

### Model verification and validation

Due to the limited availability of validation protocols for full-spine AIS models, therefore the spinal finite element model in this study was validated by comparing it with preoperative X-ray radiographs and the range of motion (ROM) of the lumbar spine in literature to verify its effectiveness. Specifically, the geometric validity of the scoliotic spine model was verified by comparing the coronal and sagittal Cobb angles measured from the model with those from anteroposterior and lateral full-length spinal radiographs. To validate the biomechanical effectiveness of the model, the sacrum was fixed, and a vertical force of 400 N was applied to the upper endplate of the L1 vertebra to simulate the upper body weight. Subsequently, a torque of 10 Nm was applied to observe the ROM of the lumbar spine during flexion, extension, lateral bending, and axial rotation, with the ROM of L1 relative to the sacrum compared to values in the literature.

### Simulation of surgical correction

To simulate the prone surgical position, the sacrum was fixed, and T1 was allowed to translate and rotate along the long axis of the spine. Diverse corrective forces were applied to the three distant models. For M1, a single rod on the concave side was subject to a 90° clockwise rotation in the apical region. For M2, both rods were concurrently rotated 90° clockwise in the apical region. For M3, both rods were simultaneously rotated 90° clockwise, with additional fixation applied to the lower instrument vertebrae and a counteracting force anticlockwise exerted to the apical vertebra, namely SDRRD. Subsequently, the three models were compared in terms of postoperative changes in coronal, sagittal and axial plane correction under different corrective forces, maximum stress in the screw-rod assembly, individual screws, and rods, as well as stress in the vertebrae and adjacent intervertebral discs within the upper instrumented vertebra region (UIV), apical vertebra region (AV), and lower instrumented vertebra region (LIV).

## Results

### Finite element model establishment and validation

A complete finite element model of AIS was established, ranging from T1 to the sacrum, including 12 thoracic vertebrae, 5 lumbar vertebrae, 1 complete sacral vertebra, and 17 intervertebral discs, along with a complete ligamentous structure. The nodes and elements of the three models are 167926 and 599626, 274764 and 1110426, and 321868 and 1281463, respectively.

**FIGURE 3:**
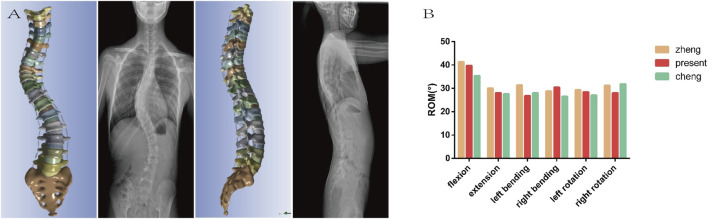
**(A)** The geometric validation indicates that the model is essentially consistent with the preoperative X-ray in both the coronal and sagittal planes. **(B)** The mechanical validation shows that the ROM of the model is basically between the values reported in the literature. Both of which concurrently confirms the effectiveness of the model.

The comparison of X-ray and model including Cobb (54.52° vs. 53.21°), AVT (36.68 mm vs. 35.39 mm), TK (30.02° vs. 29.28°), LL (−52.35° vs. −51.17°) has not revealed considerable difference. The ROM of L1 relative to the sacrum was 39.76°in flexion, 28.07°in extension, 26.83°in left lateral bending, 30.46°in right lateral bending, 28.42°in left rotation, and 28.01°in right rotation. Since the lumbar spine was in a state of left convexity, the right lateral bending was larger and the left lateral bending was smaller than those in other cases, which were between the model by Zheng et al. (2015) and the model by Chen et al. (1999). Overall, the geometric and mechanical validation of the model was confirmed to be effective (Figure 3).

### Outcomes of surgical correction

**FIGURE 4:**
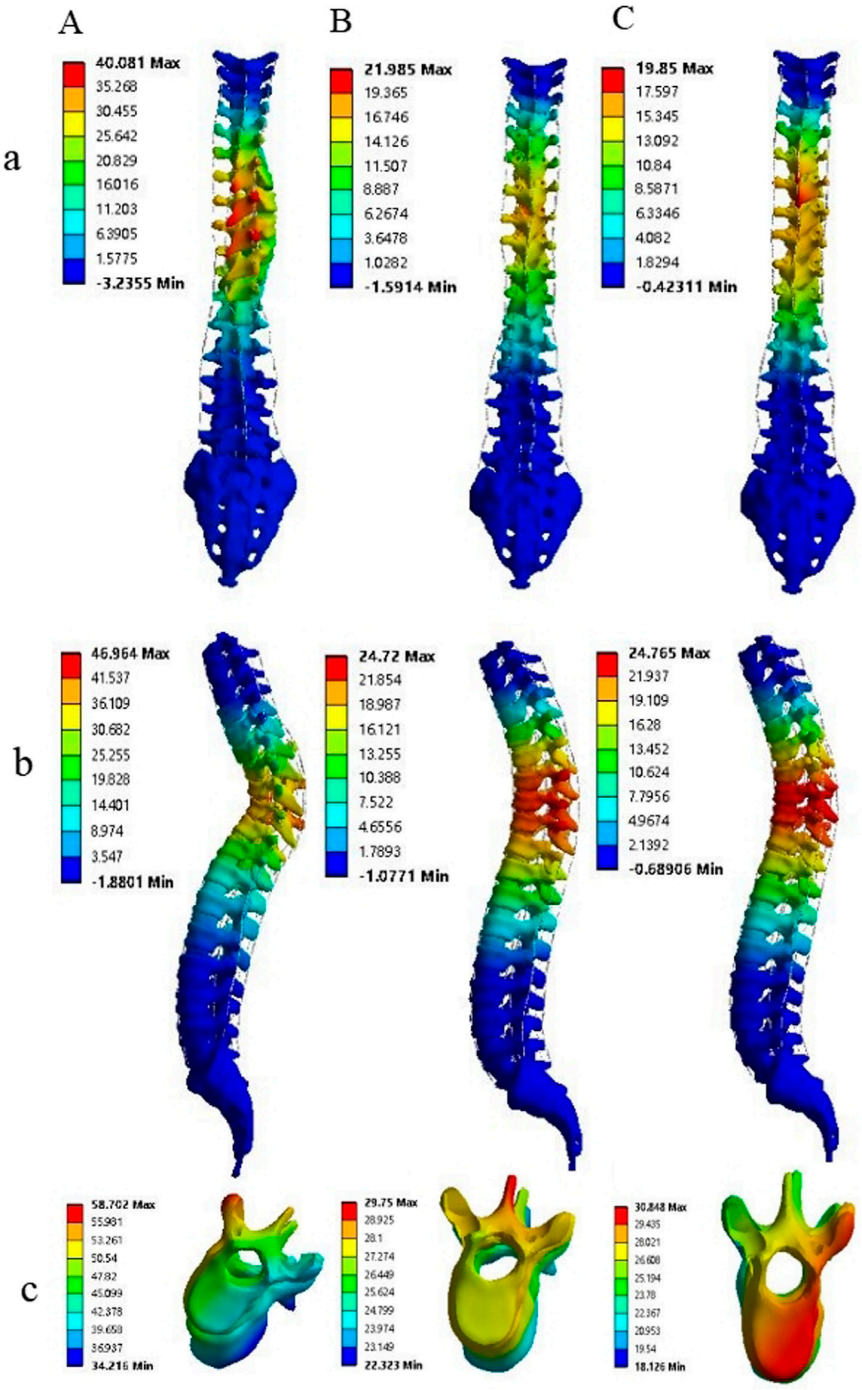
The correction outcomes of M1, M2, and M3 are respectively denoted by **(A–C)**, while the results in the coronal, sagittal, and axial planes are represented by (a–c), respectively. Comparative analysis reveals that in the coronal and sagittal planes, the correction efficacy of M2 and M3 is markedly superior to that of M1. Notably, M3 achieves the most optimal correction in axial rotation.

The comparison of coronal correction parameters revealed that Cobb angle and AVT improved in all three groups, with the best correction achieved in M3 and the worst in M1. Regarding sagittal parameters, TK exhibited kyphosis in M1, while M2 and M3 showed similar results, maintaining a better normal range. For axial rotation parameters, all three groups demonstrated improvement, with the best rotational correction in M3 and the worst in M1 (Table 2; Figure 4).

**TABLE 2:** Outcomes of corrective surgery within three models.

Parameters	X ray	M1	M2	M3
Cobb (°)	43	18.43	13.27	12.76
AVT (mm)	36.68	24.87	15.23	13.75
TK (°)	30.02	52.43	29.53	27.17
LL (°)	−52.35	−45.43	−49.87	−48.89
RAsag (°)	26.8	21.25	16.17	4.21

### Stress analysis at vertebrae and adjacent intervertebral discs after correction

**FIGURE 5:**
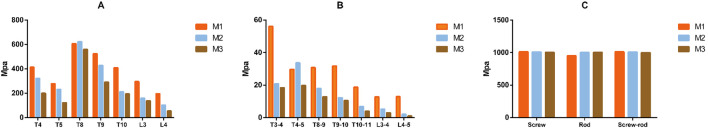
The figure illustrates that the maximum stress on the vertebrae, intervertebral disc and implants among the three models on **(A–C)**, respectively. A gradual reduction in the maximal stress experienced by the vertebrae and intervertebral discs is observed from M1 to M3, with the minimal stress level being observed in M3. Conversely, the maximal stress endured by the internal fixation devices remains largely consistent across the three models.

The maximum stress of vertebrae at UIV (T4, T5), AP (T8, T9, T10) and LIV (L3, L4) was demonstrated in Figure 5. The stress on the AP is greatest and on the LIV is lowest in the vertebrae and intervertebral discs, from M1 to M3. A considerable decrease in stress is observed from M1 to M3 in most regions, with the highest stress detected in M1 and the lowest stress seen in M3 (Figure 5A; Table 3). Compared to M1, model M3 exhibited reductions in the average maximum vertebral stress of 54%, 32.23%, and 60.79% at the UIV, AP, and LIV regions, respectively. When compared to M2, corresponding reductions of 42%, 17.25%, and 26.32% were observed in the same regions. The figure illustrates a considerable downward trend in intervertebral disc stress from M1 to M3 across the UIV(T3-4, T4-5), AP (T8-9, T9-10, T10-11), and LIV(L3-4, L4-5). Particularly, the lowest stress levels are observed in M3, with the most notable reduction seen in the LIV region (Figure 5B; Table 4). Compared to M1, model M3 exhibited a reduction in average maximum disc stress of 55.63% at the UIV, 66.46% at the AP, and 84.79% at the LIV region. Similarly, relative to M2, stress reductions of 30.17%, 26.22%, and 46.19% were observed in the UIV, AP, and LIV regions, respectively.

**TABLE 3:** Comparison of the maximum stress in different vertebrae among the three models.

	UIV		AP			LIV	
Models	T4	T5	T8	T9	T10	L3	L4
M1	414.65	278.71	607.32	525.12	409.52	295.71	196.19
M2	322.56	231.35	622.28	428.15	212.29	159.09	102.67
M3	199.44	121.53	559.06	290.9	195.01	137.78	55.08

**TABLE 4:** Comparison of the maximum stress in different intervertebral disc among the three models.

	UIV		AP			LIV	
Models	T3-4	T4-5	T8-9	T9-10	T10-11	L3-4	L4-5
M1	56.17	29.69	30.81	31.81	18.69	12.71	12.93
M2	20.84	33.71	17.91	12.23	6.83	5.28	2.08
M3	18.37	19.72	12.82	10.48	3.98	2.95	0.95

### Stress analysis at implants after correction

The figure illustrates that the maximum stress on the screws, rods, and screw-rod assembly exhibited no considerable differences among the three models (Figure 5C).

## Discussion

The surgical treatment of AIS is to correct the mechanical asymmetry of the spine through internal fixation devices, correct the deformity from a three-dimensional perspective, and reduce complications (Peng et al., 2020). However, even with the continuous development of technology and materials, there are still many surgical complications related to surgery, such as surgical incision infection, nerve injury, dural injury, internal fixation-related complications, and worsening of scoliosis (Al-Mohrej et al., 2020; Carreon et al., 2007). Bartley et al. found that the incidence of implant-related complications is second only to surgical incision infection (Bartley et al., 2017). The huge stress applied in AIS correction, acting on the implants and spine, can greatly affect the occurrence of implant-related complications. To this end, scholars have used different materials to clarify the biomechanics of the interaction between the implant and the spine during the correction process. Borkowski used frozen thoracic vertebral specimens to simulate vertebral derotation techniques, obtaining the maximum force and believed that surgeons should apply stress slowly during the correction process to avoid complications such as fractures (Borkowski et al., 2016). Cheng used thoracic vertebral specimens to compare the critical torque of failure of different internal fixation methods to verify the mechanical advantages of implants (Cheng et al., 2010). Pankowski used intact human spinal specimens to simulate the maximum torque during the process of bilateral apical vertebral rotation techniques to clarify its safety upper limit (Pankowski et al., 2019).

Since human tissue is relatively difficult to obtain, and FEA can simulate the complete spine and conduct repeated experiments, it has gradually been accepted by researchers. Aubin and Dumas used FEA to simulate rod rotation techniques and *in-situ* rod shaping techniques, respectively (Dumas et al., 2005; Aubin et al., 2003). Zhang used FEA to simulate the lumbar spine of AIS patients and found that the concave side is subjected to greater force and the intervertebral disc is subjected to the greatest force during rotation (Zhang et al., 2021). He and colleagues used FEA to verify the mechanical effectiveness of a new type of internal fixation system (He et al., 2021). An increasing number of scholars are using FEA for spinal biomechanics research, which can not only simulate normal loading conditions but also observe the stress range during correction and compare and verify different correction surgeries and implants.

Therefore, this study utilized FE analysis to simulate a novel surgical technique (M3) and compared it with traditional methods (M1 and M2) to verify its three-dimensional correction capabilities and mechanical advantages. The postoperative comparison of three-dimensional parameters revealed considerable differences in correction outcomes among the three groups. The Cobb angle in the coronal plane was smaller in the M3 and M2 groups compared to the M1 group after correction. Due to the uneven placement of screws in the finite element simulation, the connecting rod was modeled based on the screw heads through three dimensional reconstruction, rather than the smooth pre-bent rod used in actual surgery to compress the screws. Therefore, the connecting rod in the FEA may have multiple different curvatures in different planes. After rod rotation, these irregular curvatures of the rod may be transformed into spinal curvatures. The double-rod system may have inconsistent curvatures, and after rod rotation, the inconsistent curvatures can lead to force cancellation, resulting in better coronal plane correction than single rods. For this reason, the initial design of M3, which aimed to reduce the Cobb angle to achieve better TK correction, did not show a considerable difference compared to M2 but was still much better than M1. Moreover, since the finite element model was limited to the skeletal structures of the cervical, lumbar, and sacral vertebrae and did not include muscles or the rib cage, it fails to accurately simulate the biomechanical resistance against rod rotation. This results in an over-correction of TK in M1, producing a not-ideal angle than would be seen in actual surgery. In terms of axial rotation correction, the application of counteracting forces in the apical region considerably improves the axial correction in M3 compared to other models.

In terms of vertebral stress, the maximum stress in all regions was lower in Model M3 compared to Models M1 and M2. Since Model M3 involved a reduction of the Cobb angle to simulate the initial translational correction of the vertebral body by the new technique, the overall radius of the rotation rod was smaller. Additionally, a counter-rotational force was applied in the apical region, and vertebral stability was achieved in the LIV region. These factors contributed to a considerable reduction in stress on the AP and LIV regions in Model M3 compared to Models M1 and M2. During the rod rotation process, the UIV and LIV primarily experienced rotational displacement. However, the AP had a larger rotation and displacement due to the alternation of the rod on a plane can generate proximity and compression that will result in higher stress in the AP compared to the extremities. Moreover, because the torque generated by single-rod rotation is counteracted by the entire spine, the stress on the vertebral bodies is more dispersed with double rods. Therefore, the stress in Model M1 was generally higher than in Models M2 and M3. AIS patients skeletal development is not yet fully mature. According to the Hueter-Volkmann principle, areas under the highest stress can inhibit bone growth and development and bone growth can resume when the stress is relieved (Stokes, 2008). Multiple studies have also shown that asymmetry in bone mechanics is associated with AIS (Goto et al., 2003; Huynh et al., 2007). Therefore, reducing bone stress is key to correcting the pathogenesis of AIS and allowing patients to recover bone growth after surgery. Additionally, reducing stress can also decrease the risk of internal fixation failure during the correction process.

The stress analysis of intervertebral discs revealed that Model M3 exhibited reduced stress compared to Models M1 and M2. In the UIV, the vertebral bodies, being adjacent to the unfused free vertebrae, serve as a stress transfer interface. Particularly, the T3-4 intervertebral disc is subjected to considerable stress. Within the fixed segment, the stress is progressively dispersed through the connecting rod, resulting in higher stress at the contact interface compared to the fused segments. Moreover, the counter-rotational force applied along with the stabilization of the LIV in Model M3 can induce stress cancellation in the AP and LIV. Consequently, the intervertebral discs in Model M3 experience less stress than in Models M1 and M2. Intervertebral discs, consisting of the annulus fibrosus and nucleus pulposus, are essential for cushioning and transferring pressure loads, and their morphology and biomechanics change with the bony structure alterations in AIS (Guo et al., 2016). Bogdan et al. identified intervertebral disc degeneration as one of the main causes of low back pain (Costăchescu et al., 2022). Krismer et al. found that tears in the annulus fibrosus during disc degeneration are related to the rotational torque experienced (Krismer et al., 2000). All three models in this study are based on rod rotation techniques, with rotational torque applied during correction in both the fused and transitional zones between fused and unfused segments. The considerable reduction in intervertebral disc stress in Model M3, compared to Models M1 and M2, may potentially decrease disc injury and degeneration, thereby reducing the incidence of complications such as disc degeneration and low back pain.

This study has several limitations: (1) The nature of FEA means that its findings can only explain the results of the current study subject, and multiple samples are still needed for statistical analysis to enhance its generalizability; (2) This study focused on one patient with Lenke 1 CN classification and did not analyze other types. Future research could apply the findings of this study to other classifications for broader validation; (3) This finite element model, built solely from CT data, reconstructs only the spinal skeletal structures and fails to incorporate stabilizing elements such as muscle tissues and the rib cage. Consequently, the simulation results of the surgical procedure are expected to exhibit certain deviations. (4) The validity of the finite element model is limited by its exclusive numerical validation against prior models and a lack of correlation with *in vitro* biomechanical data. Moreover, as the validation was confined to the lumbar spine, the stiffness properties of the thoracic spine remain unverified. Future work should address these limitations by incorporating direct validation against *in vitro* data, particularly for the critical thoracic region.

## Conclusion

Compared with traditional corrective surgery, the Simultaneous Double-Rod Reverse Derotation technique not only achieves better correction in the coronal and sagittal planes but also has a distinct advantage in axial rotation correction, with considerably lower stress on the vertebrae and intervertebral discs.

## Data Availability

The raw data supporting the conclusions of this article will be made available by the authors, without undue reservation.
